# Edge-Embedded Multi-Feature Fusion Network for Automatic Checkout

**DOI:** 10.3390/jimaging11100337

**Published:** 2025-09-27

**Authors:** Jicai Li, Meng Zhu, Honge Ren

**Affiliations:** 1 College of Computer and Control Engineering, Northeast Forestry University, Harbin 150040, China; lijicai@nefu.edu.cn; 2 College of Information Engineering, Harbin University, Harbin 150076, China; 3 Heilongjiang Forestry Intelligent Equipment Engineering Research Center, Harbin 150040, China

**Keywords:** automatic checkout, object detection, multi-feature fusion, edge enhancement

## Abstract

The Automatic Checkout (ACO) task aims to accurately generate complete shopping lists from checkout images. Severe product occlusions, numerous categories, and cluttered layouts impose high demands on detection models’ robustness and generalization. To address these challenges, we propose the Edge-Embedded Multi-Feature Fusion Network (E2MF2Net), which jointly optimizes synthetic image generation and feature modeling. We introduce the Hierarchical Mask-Guided Composition (HMGC) strategy to select natural product poses based on mask compactness, incorporating geometric priors and occlusion tolerance to produce photorealistic, structurally coherent synthetic images. Mask-structure supervision further enhances boundary and spatial awareness. Architecturally, the Edge-Embedded Enhancement Module (E3) embeds salient structural cues to explicitly capture boundary details and facilitate cross-layer edge propagation, while the Multi-Feature Fusion Module (MFF) integrates multi-scale semantic cues, improving feature discriminability. Experiments on the RPC dataset demonstrate that E2MF2Net outperforms state-of-the-art methods, achieving checkout accuracy (cAcc) of 98.52%, 97.95%, 96.52%, and 97.62% on Easy, Medium, Hard, and Average mode, respectively. Notably, it improves by 3.63 percentage points in the heavily occluded Hard mode and exhibits strong robustness and adaptability in incremental learning and domain generalization scenarios.

## 1. Introduction

With the rapid advancements in deep learning and computer vision, Automatic Checkout (ACO) technology has emerged as a key driver for intelligent transformation in the retail industry. ACO leverages computer vision to analyze product images in real time and automatically generate shopping lists. This approach overcomes the efficiency bottlenecks of manual checkout and supports the development of unmanned retail and personalized services. Its advances carry both strong theoretical significance and broad practical value.

However, ACO faces several technical challenges such as product occlusion, overlap, diverse poses, and high inter-class similarity. These difficulties demand stronger structural understanding, boundary perception, and fine-grained feature modeling.

**Figure 1:**
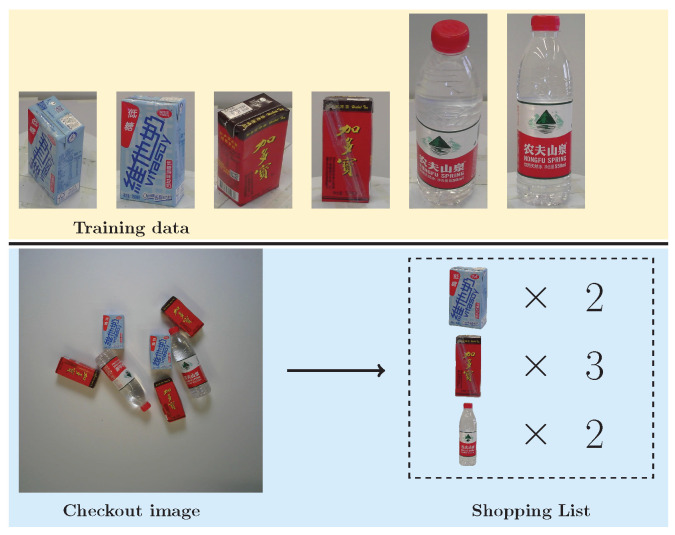
Illustration of the Automatic Checkout (ACO) task and its domain gap. The ACO task aims to generate complete shopping lists by detecting all products in checkout images. However, training data typically consists of multi-view images of single isolated products, while test data (checkout images) involves real-world scenes with multiple occluded and overlapping items, leading to significant cross-domain differences that hinder model generalization.

Furthermore, as illustrated in Figure 1, ACO faces a significant domain gap: training data often consists of multi-view images of individual products, while test data comprises real-world checkout images containing multiple items. This discrepancy in data distribution hinders direct model generalization. In addition, the frequent update of product categories in retail scenarios introduces further challenges for model scalability. Consequently, the ACO task is essentially a complex problem of cross-domain fine-grained object detection and counting.

Existing research addresses these challenges mainly in two directions. The first is synthetic data generation, using methods such as cut-and-paste and style transfer. This alleviates data scarcity and supports cross-domain learning. However, synthetic samples often distort physical structures and fail to capture realistic layouts and occlusion patterns. As a result, a domain gap remains, which limits model generalization. The second direction focuses on strengthening feature representation. This improves detection robustness under occlusion, fine-grained categories, and complex layouts. Moreover, most detection frameworks are still designed for general tasks and lack the capacity to model edge features and structural details. In dense and heavily occluded checkout scenes, they often fall short of the accuracy required in retail applications.

To address these challenges, we propose a unified framework for data augmentation and structural modeling, called Edge-Embedded Multi-Feature Fusion Network (E2MF2Net). On the data side, we design a Hierarchical Mask-Guided Composition (HMGC) strategy that incorporates pose pruning and occlusion control mechanisms to ensure physical plausibility and structural realism of synthesized images. Additionally, mask-based supervision is introduced to enhance the model’s perception of boundaries and occluded regions. On the structural side, we construct an Edge-Embedded Enhancement Module (E3) and a Multi-Feature Fusion Module (MFF) to improve the model’s capability in structural modeling and semantic representation. Extensive experiments on the RPC dataset, including systematic ablation studies and incremental learning evaluations, demonstrate the effectiveness of our method in addressing challenges such as complex occlusions, cross-domain generalization, and category expansion.

In summary, the main contributions of this work are as follows:

- We propose E2MF2Net, a novel detection framework for ACO tasks, which achieves collaborative optimization in image synthesis and feature modeling, effectively alleviating the insufficient detection accuracy of mainstream methods in complex occlusion scenarios.
- A Hierarchical Mask-Guided Composition (HMGC) strategy based on pose stability and occlusion tolerance is designed to enhance the structural consistency and perceptual realism of synthetic images.
- An Edge-Embedded Enhancement Module (E3) and a Multi-Feature Fusion Module (MFF) are constructed to respectively enhance the model’s boundary perception and multi-scale semantic integration capabilities.
- The proposed method achieves leading performance on the RPC dataset and demonstrates strong generalization and robustness in scenarios such as incremental learning and complex occlusion.

## 2. Related Work

### 2.1. General Object Detection Frameworks

Object detection methods can be broadly categorized into two paradigms: one-stage and two-stage approaches. One-stage detectors, such as the YOLO [1,2] series, directly perform classification and regression on feature maps in a single forward pass, offering fast inference speed and suitability for real-time applications. In contrast, two-stage detectors, such as the R-CNN [3,4] family, decompose the detection pipeline into region proposal generation followed by classification and regression, achieving higher accuracy, especially in tasks involving severe occlusions or requiring fine-grained recognition.

Regardless of the detection paradigm, most frameworks adopt a Backbone-Neck-Head architecture. The Backbone (e.g., VGG16 [5], ResNet [6], MViT [7], MViTv2 [8]) serves as the fundamental module for visual feature extraction, providing low-level semantic representations. Notably, MViTv2 employs spatial downsampling, multi-head self-attention, and attention pooling. These mechanisms enhance object boundary localization and contextual modeling, which leads to strong performance in ACO tasks.

The Neck, such as the Feature Pyramid Network (FPN) [9], enhances multi-scale feature fusion to improve the detector’s sensitivity to objects of various sizes. The Head is responsible for final prediction tasks. For example, Cascade R-CNN [10] introduces multi-stage refinement mechanisms at the Head level, significantly boosting robustness and accuracy in complex scenes.

Recent studies have improved general object detection frameworks through lightweight design and feature optimization, enabling applications in hyperspectral classification [11], change detection [12], and small-object recognition [13]. Multimodal fusion and structural innovations have also been widely applied to autonomous driving [14] and railway defect detection [15], achieving efficient detection under limited computational resources. In addition, the integration of transformers and convolutions has advanced salient object detection and remote sensing segmentation, while cross-scale modeling further enhances model generalization [16,17,18,19].

### 2.2. Feature Representation and Structural Enhancement

To enhance the adaptability of detectors to multi-scale objects and complex scenes, extensive research has been conducted. A major line of work emphasizes the structural optimization of the Neck module. In particular, most studies focus on improving feature fusion and strengthening structural representation.

In terms of feature fusion, FPN and its variants (e.g., PANet [20], BiFPN [21]) enhance feature representation by exploring multi-scale feature fusion methods. Some studies [22,23,24,25] attempt to integrate multi-scale 2D feature maps into a unified high-dimensional space. This effectively constructs a “pseudo-3D” feature structure that models spatial layout, channel dependencies, and semantic content together. As a result, the contextual representation capabilities of the network are enhanced.

For structural enhancement, edge-aware mechanisms have been widely employed to strengthen the model’s ability to perceive object boundaries and structural details. Some works [26,27] explicitly guide the network to model object contours by fusing low-level edge details with high-level semantics, thus improving both structural awareness and detection accuracy. Other studies [28,29,30] have also proposed multi-level pooling strategies to explicitly extract structural boundary features.

Despite the notable progress in multi-scale semantic integration, current feature fusion approaches still fall short in modeling structural boundaries explicitly—particularly when dealing with fine-grained variations or heavy occlusions. Meanwhile, although edge enhancement methods improve the detector’s sensitivity to boundary cues, their integration with multi-scale semantic features remains suboptimal. Motivated by these observations, we propose a structural enhancement strategy that combines multi-feature fusion and edge awareness to improve the model’s capacity for complex structural modeling, thereby better addressing the fine-grained recognition and localization challenges inherent in ACO tasks.

### 2.3. Automatic Checkout Systems

With the continuous advancement of research in product visual recognition, a wide range of public datasets has been developed to support model training and evaluation across various application scenarios. However, significant differences exist among these datasets in terms of task suitability, scene complexity, and data distribution, making them difficult to apply directly to ACO tasks.

Early grocery-related datasets—such as SOIL-47 [31], Freiburg Groceries Dataset [32], SHORT [33], MVTec D2S [34], and Supermarket Produce Dataset [35]—are small and mainly support single-object classification, making them unsuitable for complex checkout scenarios. Shelf or warehouse datasets like GroZi-120 [36], Grocery Products Dataset [37], RP2K [38], SKU-110K [39], and Locount Dataset [40] feature orderly layouts, which differ from cluttered checkout scenes. Datasets for vending machines (UVMs [41], TGFS [42]) have narrow applicability, while large-scale e-commerce datasets (Products-6K [43], AliProducts [44], Products-10K [45], Product1M [46]) focus on retrieval or text-image matching and lack occlusion modeling.

These limitations motivate the use of datasets specifically designed for complex checkout scenarios. In contrast, the Retail Product Checkout (RPC) [47] dataset provides large-scale, richly annotated checkout images with natural stacking and occlusions, closely mimicking real cashier environments. Its high representativeness makes it the primary benchmark for ACO model training and evaluation in this study.

To address the challenges in ACO scenarios, various methods have been proposed based on the RPC dataset. Wei et al. [47] established a baseline using copy-pasted synthetic images and CycleGAN-based [48] adaptation. Li et al. [49] introduced a posture filtering method and DPNet for joint detection and counting. IncreACO [50] improved synthesis by incorporating real spatial layouts. DPSNet [51] extended DPNet with multi-scale feature learning and iterative distillation. Sun et al. [52] proposed CGFENet, integrating global and local attention into FPN. PSP [53] built prototype representations for similarity-based discrimination, followed by PLACO [54] which aligned cross-domain prototypes. S2MC2Net [55] used point-level supervision and density maps for counting, iCashier [56] introduced triplet learning against forgetting, and DEGNet [57] adopted a decoupled edge-image architecture for improved localization.

In addition, some recent works [58,59,60] have explored the adaptation or fine-tuning of YOLO series detectors to facilitate lightweight ACO deployment on edge devices. Recent advances in multi-view learning for handling incomplete multi-modal data [61,62] offer new perspectives for addressing the challenges of missing or conflicting multi-sensor data in ACO systems. While previous methods have partially addressed domain gap and occlusions, most still face limitations in synthesizing high-quality data and lack mechanisms for jointly modeling edge structures and multi-scale semantics. These shortcomings hinder the effectiveness of current approaches in challenging scenarios. Consequently, there remains considerable room for improving recognition accuracy and enhancing model generalization.

## 3. Methodology

### 3.1. Hierarchical Mask-Guided Composition

To address the limitations of existing synthetic product image generation methods in terms of spatial layout and structural consistency, a Hierarchical Mask-Guided Composition (HMGC) strategy is proposed. This method selects product instances with more natural and realistic poses by analyzing mask compactness, and incorporates pose pruning and structural control mechanisms to generate virtual checkout images that conform better to physical constraints and real-world scene structures.

Furthermore, we design a mask-supervised training pipeline to explicitly guide the model in perceiving object boundaries and spatial structures, thereby enhancing its robustness to the distribution gap between synthetic and real images (see Section 4.3 for details). The overall HMGC strategy comprises two key components: one is a pose pruning strategy based on mask compactness, which eliminates unnatural product placements; the other is a mask-aligned image composition and supervision mechanism, which enables high-quality image generation and promotes structural consistency learning.

#### 3.1.1. Pose Pruning Strategy Based on Mask Compactness

For every product category c∈C, let the corresponding image set be $I_{c}=\left\{I_{c}^{1},I_{c}^{2},\ldots ,I_{c}^{n}\right\}$, where each item is captured from four viewpoints with multiple in-plane rotations. Background pixels are removed using the method in DPNet [49]. However, not every pose is physically plausible in real checkout scenarios—for example, hanging snack bags in a vertical pose or upside-down beverage bottles are rarely observed in practice. Consequently, eliminating pose-anomalous samples before composition is a necessary preprocessing step. To this end, we introduce a stability metric that combines mask area and boundary compactness to evaluate the geometric plausibility and visual usability of each instance. Specifically, for a single product image *I*, the non-zero region of its alpha mask is extracted, and the minimum area enclosing rotated rectangle R(I) is computed. The Mask Rect Rate (MRR) of *I* is defined as MRR(I)

(1) $$\mathrm{MRR}\left(I\right)=\frac{\left|Mask(I)\right|}{\mathrm{Area}(R(I))},$$

where Mask(I) is the foreground-mask pixel set, |Mask(I)| is its cardinality (mask area) and Area(R(I)) denotes the area of the enclosing rectangle. When MRR(I)→1, the product boundary is compact and the pose is considered stable and suitable for synthesis. For any $I_{c}^{i}\in I_{c}$, we further define its Stability Score as $S_{\mathrm{stable}}\left(I_{c}^{i}\right)$

(2) $$S_{\mathrm{stable}}\left(I_{c}^{i}\right)=\frac{\left|Mask(I_{c}^{i})\right|}{Mask_{c}^{\max}}\cdot \frac{\mathrm{MRR}(I_{c}^{i})}{{\mathrm{MRR}}_{c}^{\max}},$$

where $Mask_{c}^{\max}=\max_{I_{c}^{i}\in I_{c}}\left|Mask\left(I_{c}^{i}\right)\right|$ is the maximum mask area in category *c* and ${\mathrm{MRR}}_{c}^{\max}=\max_{I_{c}^{i}\in I_{c}}\mathrm{MRR}\left(I_{c}^{i}\right)$ is the maximum compactness. The first term measures relative mask size within the category, and the second the relative compactness. All stability scores are clustered with Otsu’s method [63] to obtain an adaptive threshold α (set to 0.6 in this work). Images with $S_{\mathrm{stable}}\left(I_{c}^{i}\right)\geq \alpha$ are retained as high-quality samples for subsequent composition.

In real checkout scenes, inter-product occlusions depend not only on spatial position but also on geometric and structural constraints. For example, a flimsy bag cannot realistically rest horizontally on top of an upright bottle. To mimic such physical realism, we define an Occlusion Tolerance (OT) for each category *c* as

(3) $$\mathrm{OT}\left(c\right)=0.8\sqrt{\frac{\max(0,\beta (c)-0.5)}{2.5}},$$

where the parameter β(c) is computed by

(4) $$\beta \left(c\right)=\frac{Mask_{\mathrm{top}}^{\min}\left(c\right)}{Mask_{\mathrm{horizontal}}^{\max}\left(c\right)},$$

with $Mask_{\mathrm{top}}^{\min}\left(c\right)$ the minimal mask area in the top-view, and $Mask_{\mathrm{horizontal}}^{\max}\left(c\right)$ the maximal mask area in the horizontal view. When β(c) is larger, it indicates that the category exhibits similar shapes across viewpoints and can physically sustain a certain degree of occlusion or stacking; a smaller β(c) indicates lower tolerance to being occluded.

#### 3.1.2. Image Composition Strategy

After pose pruning and sample filtering, we design a structured multi-stage image composition pipeline to paste multiple preprocessed single-product images onto a shared background, thereby generating checkout images with realistic occlusion patterns and geometrically plausible layouts. This process involves a series of position sampling steps, occlusion constraints, and paste scheduling rules to ensure that the resulting images are both visually realistic and structurally annotatable.The complete procedure of this strategy is summarized in the pseudocode provided in Algorithm 1.

Formally, let the set of composable elements be $Syn=\{Syn_{1},Syn_{2},\ldots ,Syn_{n}\}$, the background image be $Syn_{b}$, and the final composite image be $I_{syn}$. We first sort Syn in descending order according to each item’s OT. Each item is augmented before being pasted, using random rotation (in the range $\left[-180^{\circ },180^{\circ }\right]$) and scaling (scaling factor in [0.4,0.7]) to increase diversity. Next, the first element $Syn_{1}$ is pasted onto $Syn_{b}$ using its foreground mask region to generate the initial composite image $I_{syn}^{1}$. Its pasted region is recorded as mask $M_{1}$, and the initial mask set is $M=\{M_{1}\}$. For each subsequent element $Syn_{i}\;\left(i>1\right)$, the following strategy is applied

- Candidate Pasting Point Extraction via Edge Density:
  - Compute the union of existing masks $M_{\mathrm{union}}^{i-1}={⋃}_{j=1}^{i-1}M_{j}$;
  - Extract edge pixels from the union mask using the Sobel operator $P_{\mathrm{edge}}^{i-1}=\mathrm{Edge}\left(M_{\mathrm{union}}^{i-1}\right)$;
  - For each edge pixel $P\left(x,y\right)\in P_{\mathrm{edge}}^{i-1}$, calculate the non-zero pixel density d(P(x,y)) within a square neighborhood N(x,y) of size k×k (e.g., *k*=200), where I[·] denotes the indicator function, which equals 1 if the condition is satisfied and 0 otherwise.
    (5) $$d(P(x,y))=\frac{1}{k^{2}}\sum_{\left(i,j)\in N(x,y\right)}I\left[M_{\mathrm{union}}^{i-1}\left(x,y\right)>0\right].$$
  - Select low-density points $d\left(P\left(x,y\right)\right)<\tau_{d}$ (threshold set to 0.5) to form the candidate paste point set $P_{\mathrm{candidate}}^{i-1}$ sorted in ascending order of density.
**Algorithm 1** HMGC: Image Composition Strategy 1:**Input:** 2:   Syn: Composable elements sorted in descending order by OT value 3:   $Syn_{b}$: Background image 4:   $\tau_{d}$: Edge density threshold (e.g., 0.5) 5:   *k*: Neighborhood size for density calculation (e.g., 200) 6:   $N_{\max}$: Maximum paste attempts per item (e.g., 500) 7:**Output:** 8:   $I_{syn}$: Generated synthetic checkout image 9:   M: Set of instance masks for $I_{syn}$10:// Initialize with first element11:$I_{syn}^{1}\leftarrow \mathrm{Paste}\left(Syn_{b},Syn_{1}\right)$12:$M_{1}\leftarrow \mathrm{GetPastedRegion}\left(I_{syn}^{1}\right)$13:$M\leftarrow \{M_{1}\}$14:**for** i←2 to |Syn| **do**15:    $Syn_{i}\leftarrow \mathrm{RandomRotateAndScale}\left(Syn_{i}\right)$16:    $\left(mask_{\mathrm{target}},I_{\mathrm{target}}\right)\leftarrow Syn_{i}$17:    success←False18:    attempts←019:    // Find suitable paste location20:    $M_{\mathrm{union}}^{i-1}\leftarrow {⋃}_{j=1}^{i-1}M_{j}$21:    $P_{\mathrm{edge}}^{i-1}\leftarrow \mathrm{Edge}\left(M_{\mathrm{union}}^{i-1}\right)$22:    $P_{\mathrm{candidate}}^{i-1}\leftarrow \mathrm{FindLowDensityPointsAndSort}\left(P_{\mathrm{edge}}^{i-1},M_{\mathrm{union}}^{i-1},\tau_{d},k\right)$23:    **while** not success **and** $attempts<N_{\max}$ **do**24:        **if** $P_{\mathrm{candidate}}^{i-1}$ is not empty **then**25:           $p_{attempts}\leftarrow \mathrm{SelectPointByIndex}\left(P_{\mathrm{candidate}}^{i-1}\right)$26:           $p_{r}\leftarrow \mathrm{SampleNearbyPoint}\left(p_{attempts}\right)$27:           // Align and paste item28:           $M_{k}\leftarrow \mathrm{FindSourceMask}\left(M,p_{attempts}\right)$29:           $I_{\mathrm{candidate}}\leftarrow \mathrm{AlignAndPaste}\left(I_{syn}^{i-1},I_{\mathrm{target}},M_{k},mask_{\mathrm{target}},p_{r}\right)$30:           $M_{i}\leftarrow \mathrm{GetPastedRegion}\left(I_{\mathrm{candidate}}\right)$31:           // Validate occlusion constraints32:           **if** $\mathrm{ValidateOcclusion}(M_{i},M)\;()$ **then**33:               $I_{syn}^{i}\leftarrow I_{\mathrm{candidate}}$34:               $M\leftarrow M\cup \{M_{i}\}$35:               success←True36:           **end if**37:        **end if**38:        attempts←attempts+139:    **end while**40:**end for**41:**return** $I_{syn},M$
- Geometric Alignment-Based Pasting:
  - Choose the first candidate point $P_{0}\in P_{\mathrm{candidate}}^{i-1}$, and randomly sample a paste reference point $P_{r}$ within a 50-pixel neighborhood;
  - Let $M_{k}\in M$ be the mask containing $P_{0}\in M_{k}$. Compute its minimum enclosing rotated rectangle with corner set $Rect_{\mathrm{source}}=\left\{R_{0}^{s},R_{1}^{s},R_{2}^{s},R_{3}^{s}\right\}$ (in counter-clockwise order), where $R_{0}^{s}$ is the bottom-left vertex.
  - Similarly, compute the rectangle for $Syn_{i}$: $Rect_{\mathrm{target}}=\left\{R_{0}^{t},R_{1}^{t},R_{2}^{t},R_{3}^{t}\right\}$
  - Identify the two vertices $\left\{R_{a}^{s},R_{b}^{s}\right\}\left(a,b\in \left\{0,1,2,3\right\}\right)$ of $Rect_{\mathrm{source}}$ closest to $P_{r}$, then randomly select a vertex $R_{c}^{t}\in Rect_{\mathrm{target}}$ such that c∉{a,b}. Align $R_{c}^{t}$ to $P_{r}$, and paste $Syn_{i}$ onto $I_{syn}^{i-1}$ accordingly, yielding candidate composite $I_{syn}^{i}$.
- Occlusion Ratio Constraint Validation:
  - Let $M_{i}$ be the pasted region of $Syn_{i}$ in $I_{syn}^{i}$. For any previous mask $M_{j(j<i)}\in M$, define its top-layer union as $M_{\mathrm{top}-\mathrm{union}}^{j}=\left({⋃}_{k=j+1}^{i-1}M_{k}\right)\cup M_{i}$; Then, the occlusion ratio of $M_{j}$ is $r_{\mathrm{shade}}\left(M_{j}\right)$
    (6) $$r_{\mathrm{shade}}\left(M_{j}\right)=\frac{|M_{j}\cap M_{\mathrm{top}-\mathrm{union}}^{j}|}{|M_{j}|}.$$
  - If $r_{\mathrm{shade}}>0.5$, the paste is invalid; proceed to the next candidate point in $P_{\mathrm{candidate}}^{i-1}$ and repeat. If all attempts (max 500) fail, discard $Syn_{i}$.
  - If occlusion constraints are satisfied, add $M_{i}$ to M and update $I_{syn}^{i}$. Proceed to the next item.
- Final Supervision and Annotation Generation: By enforcing OT-based priority and progressive occlusion validation, we ensure that occlusion-sensitive items appear on top, while geometrically stable items act as structural support. A dynamic supervision strategy ensures that the occlusion ratio of any object in the final virtual image remains within a tolerable range. After all elements in Syn are successfully composed, we obtain a structurally compact and realistic image $I_{syn}$ and its mask set M. The set M enables the generation of annotations compatible with the RPC dataset. Compared to the RPC test set, which only includes bounding boxes and center-point annotations, our pipeline provides instance-level masks, offering stronger structural supervision.

### 3.2. Architecture of the Detection Framework

**Figure 2:**
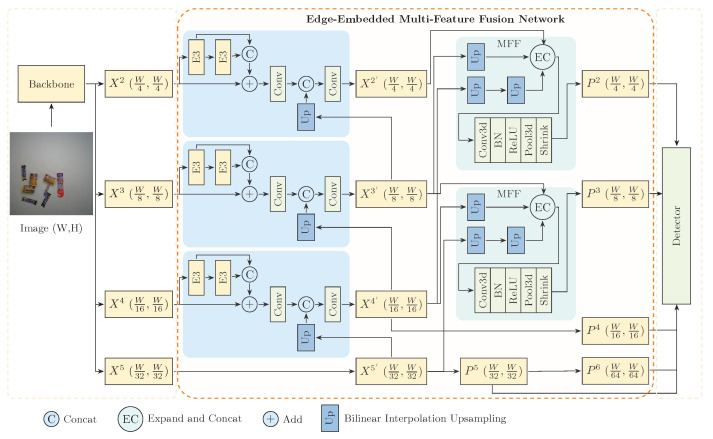
Architecture of the detection framework within the proposed Edge-Embedded Multi-Feature Fusion Network (E2MF2Net). It contains two modules: the Edge-Embedded Enhancement Module (E3) for boundary-aware feature enhancement and the Multi-Feature Fusion Module (MFF) for multi-scale semantic fusion.

As part of the proposed Edge-Embedded Multi-Feature Fusion Network (E2MF2Net), the detection framework is designed to effectively capture fine-grained product features in complex ACO scenarios. This framework primarily comprises two key modules: the Edge-Embedded Enhancement Module (E3) and the Multi-Feature Fusion Module (MFF), as shown in Figure 2.

Specifically, the E3 module enhances each feature level with top-down edge-guided processing, embedding salient structural cues into local feature maps to strengthen the representation of object boundaries. Building on this, the MFF module enhances the contextual modeling of semantic features while preserving edge-aware cues, ultimately producing more discriminative feature maps for prediction by the detection head.

#### 3.2.1. Edge-Embedded Enhancement Module

The proposed E3 aims to overcome the limitations of conventional FPN architectures in preserving low-level structural cues—particularly their deficiency in representing object boundaries, contours, and local transitions. While standard top-down pathways in FPNs effectively propagate semantic context, they often dilute spatially detailed information from shallow layers, which is critical for fine-grained object localization under occlusions and background clutter.

As illustrated in Figure 2, the E3 module constructs an auxiliary enhancement path that independently processes each stage’s features to extract edge-aware representations. The design is implemented as follows.

Let the output feature map of the *i*-th stage from the backbone be denoted as $X^{i}$

(7) $$X^{i}\in R^{B\times \frac{H}{2^{i}}\times \frac{W}{2^{i}}},\;i\in \left\{2,3,4,5\right\},$$

where *B* is the batch size, and H,W are the height and width of the input image. To amplify structural cues, the edge-enhanced feature map $X_{\mathrm{edge}}^{i}$ is formulated as

(8) $$X_{\mathrm{edge}}^{i}=\mathrm{Concat}\left(\left[E3\left(X^{i}\right),E3\left(E3\left(X^{i}\right)\right)\right]\right)+X^{i},$$

where Concat(·) denotes channel-wise concatenation, and E3(·) denotes the E3 block for edge enhancement. Each E3(·) performs salient edge extraction and adaptive fusion, formulated as

(9) E3(X)=X+Att((X−AP(X)),(X−MP(X))),

where AP(·) and MP(·) denote 3 × 3 average pooling and max pooling operations with stride 1, respectively. The subtraction from the original feature map emphasizes local edge transitions by suppressing uniform regions, thus highlighting spatial discontinuities. The differential features from average and max pooling are then adaptively fused via Att(a,b)

(10) Att(a,b)=γa+(1−γ)b,

where γ∈[0,1] is a learnable attention weight that dynamically balances the fusion of differential edge responses from average and max pooling. The core idea of this design lies in performing multi-scale local context comparisons over the original feature map to enhance activations in edge transition regions. By stacking multiple E3 operations, the network expands its receptive field for edge-aware representations, thereby enriching boundary features at higher semantic levels. This attention mechanism offers a flexible way to preserve both soft contour transitions and hard edge boundaries, depending on content-specific spatial statistics.

The theoretical motivation behind this design lies in local contrastive filtering, where edge evidence is derived through contextual comparisons across multiple scales. Unlike conventional high-pass filters, which may amplify noise, the dual-path differential pooling coupled with learnable fusion adaptively selects discriminative transitions, enabling the network to retain structural consistency without sacrificing semantic abstraction.

To integrate edge-aware information into the multi-level FPN hierarchy, the enhanced features $X_{\mathrm{edge}}^{i}$ are fused with the upsampled output from the higher-level semantic features

(11) $$X^{i^{\prime }}=\mathrm{Concat}\left(X_{\mathrm{edge}}^{i},\mathrm{UP}\left(X^{{\left(i+1\right)}^{\prime }}\right)\right),\;i\in \left\{2,3,4\right\},$$

where UP(·) denotes bilinear upsampling with a scale factor of 2. $X^{5^{\prime }}$ (equivalent to $X^{5}$) is the final high-level output from the backbone. This preserves the standard top-down semantic propagation while incorporating multi-scale edge information.

**Figure 3:**
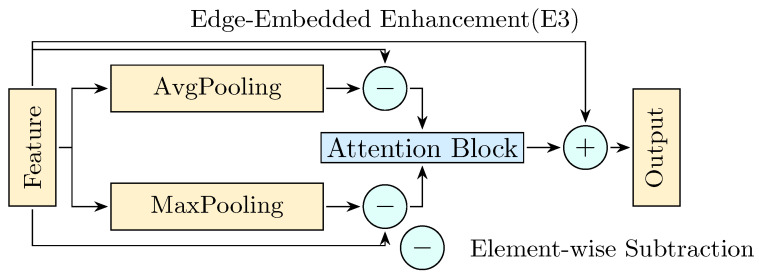
Architecture of Edge-Embedded Enhancement Module (E3). The module extracts salient edge features via differential pooling and applies attention-based fusion to enhance boundary-aware feature representations.

As illustrated in Figure 3, the E3 module forms a structurally aware multi-scale fusion loop. By reinforcing edge cues across hierarchical levels, this mechanism augments the saliency of detailed information within feature maps, thereby mitigating its dilution during top-down residual propagation. Consequently, this module strengthens the network’s capability in delineating object boundaries, disambiguating overlapping instances, and enhancing spatial coherence—factors that are crucial for detection performance, particularly in dense and occluded scenes.

#### 3.2.2. Multi-Feature Fusion Module

While the E3 module effectively enhances edge-awareness in the feature hierarchy, the network may still encounter challenges in detecting objects under complex scenes due to limited semantic abstraction—particularly in the presence of background clutter or occlusions. To address this, we propose MFF, which aims to incorporate rich contextual semantics via multi-scale integration and hierarchical compression. The core intuition is to enable fine-to-coarse feature guidance, where lower-level details and higher-level semantics are jointly optimized to strengthen both localization and category-level discrimination.

We define the feature set output from the E3 module as $X=\{X^{2^{\prime }},X^{3^{\prime }},X^{4^{\prime }},X^{5^{\prime }}\}$. Our goal is to synthesize a unified feature representation $P^{i}$ for each level, preserving low-level precision while enriching high-level semantics.

To this end, for lower stages i∈{2,3}, we perform multi-scale upsampling and feature compression through a recursive top-down fusion scheme

(12) $$P^{i}=PU\left(EC\left(X^{i^{\prime }},\mathrm{UP}\left(X^{{\left(i+1\right)}^{\prime }}\right),\mathrm{UP}\left(\mathrm{UP}\left(X^{{\left(i+2\right)}^{\prime }}\right)\right)\right)\right),$$

Here, EC(a,b,c) expands and concatenates features across three adjacent levels, enabling hierarchical cross-talk among levels and preserving contextual richness

(13) EC(a,b,c)=[a:b:c],

and PU(·) represents a lightweight 3D convolutional compression unit, composed of a 1 × 1 × 1 3D convolution, normalization, activation, spatial pooling, and dimensionality reduction. This module acts as an inter-scale context projector, learning joint correlations across spatial and scale dimensions and enhancing cross-level semantic continuity.

For higher layers in X, which already encode sufficient abstraction, we apply conventional convolutional refinement

(14) $$P^{4}={\mathrm{Conv}}_{3\times 3}\left(X^{4^{\prime }}\right),$$

(15) $$P^{5}={\mathrm{Conv}}_{3\times 3}\left(X^{5^{\prime }}\right),$$

(16) $$P^{6}={\mathrm{Conv}}_{3\times 3}\left({\mathrm{MaxPool}}_{3\times 3,s=2}\left(P^{5}\right)\right).$$

The final multi-scale feature set is $P=\{P^{2},P^{3},P^{4},P^{5},P^{6}\}$, which is passed to the detection head for final prediction.

The MFF module is grounded in feature-scale disentanglement and multi-resolution consistency. By explicitly modeling inter-scale dependencies through hierarchical fusion, the module enables the network to reconcile spatial accuracy with semantic abstraction. Unlike standard additive FPN fusion, our EC-PU scheme provides nonlinear context transformation and semantic continuity, yielding greater robustness in dense or occluded scenes.

## 4. Experiments

### 4.1. Dataset Details

To systematically evaluate the proposed image composition strategy and detection model in the context of ACO, we adopt the most representative and challenging public benchmark—the RPC dataset.

This dataset contains 200 retail product categories and 83,739 images, including 53,739 single-product images and 30,000 checkout images. The single-product images are captured under controlled lighting and backgrounds from various viewpoints, while the checkout images simulate realistic cashier scenes with diverse object arrangements, such as rotations, overlaps, and occlusions. Among the 30,000 real checkout images, 6000 are selected for auxiliary training, while the remaining 24,000 are used for performance evaluation. Furthermore, the dataset provides rich annotations (including shopping lists, point-level positions, and bounding boxes), and categorizes checkout scenes into three occlusion difficulty levels—easy (3–10 instances), medium (10–15 instances), and hard (15–20 instances). Additionally, we utilize the SKU-110K [39] dataset to further validate the model’s performance across diverse benchmarks. This large-scale dataset is characterized by densely packed retail products on shelves and contains over 11,000 images with bounding box annotations.

To address the domain gap between single-product and checkout images, we employ the proposed HMGC strategy to generate 46,000 high-fidelity synthetic checkout images for training with a fixed resolution of 1815 × 1815 pixels. This greatly enhances the diversity and generalization capacity of the training data. A summary of the datasets is shown in Table 1.

### 4.2. Evaluation Metrics

To comprehensively assess model performance in the ACO task, we utilize a set of specialized metrics defined by the RPC [47] dataset, including Checkout Accuracy (cAcc), Average Counting Distance (ACD), Mean Category Counting Distance (mCCD), and Mean Category Intersection over Union (mCIoU). These are formally defined as follows.

cAcc evaluates whether the predicted shopping list (both category and quantity) is completely correct for each image. As the most critical metric for ACO systems, higher cAcc indicates better commercial feasibility. It is computed as

(17) $$\mathrm{cAcc}=\frac{1}{N}\sum_{i=1}^{N}\delta \left(\sum_{k=1}^{K}\left|{\hat{y}}_{i,k}-y_{i,k}\right|,0\right),$$

where *N* is the number of images, *K* is the total number of product categories, ${\hat{y}}_{i,k}$ and $y_{i,k}$ denote the predicted and ground-truth count for category *k* in image *i*, respectively. The indicator function δ(a,b) = 1 if a=b, and 0 otherwise.

ACD measures the total absolute count error per image across all categories

(18) $$\mathrm{ACD}=\frac{1}{N}\sum_{i=1}^{N}\sum_{k=1}^{K}\left|{\hat{y}}_{i,k}-y_{i,k}\right|.$$

mCCD computes the average relative error per category across the dataset

(19) $$\mathrm{mCCD}=\frac{1}{K}\sum_{k=1}^{K}\frac{\sum_{i=1}^{N}\left|{\hat{y}}_{i,k}-y_{i,k}\right|}{\sum_{i=1}^{N}y_{i,k}}.$$

mCIoU measures the similarity between predicted and ground-truth shopping lists in a set-wise manner

(20) $$\mathrm{mCIoU}=\frac{1}{K}\sum_{k=1}^{K}\frac{\sum_{i=1}^{N}\min\left({\hat{y}}_{i,k},y_{i,k}\right)}{\sum_{i=1}^{N}\max\left({\hat{y}}_{i,k},y_{i,k}\right)}.$$

In addition to these ACO-specific classification and counting metrics, we also report mAP50 and mmAP following the COCO [64] evaluation protocol, to assess detection quality in terms of object localization and bounding box accuracy.

### 4.3. Implementation Details

The proposed detection network is implemented based on Detectron2 [65], with all training and inference conducted using PyTorch [66] 2.3.0 and CUDA 11.8. All experiments are run on a workstation equipped with an NVIDIA RTX 4090 GPU, with Automatic Mixed Precision (AMP) enabled to fully exploit Tensor Core acceleration. The overall architecture adopts the Cascade Mask R-CNN detection framework with MViTv2 as the backbone. The AdamW optimizer and step-based learning rate decay are used, with decay to 0.1 and 0.01 of the original rate at 0.6 and 0.9 of total iterations.

To bridge the significant appearance and distribution gap between synthetic and real checkout images, we propose a mask-supervised two-stage training paradigm (pretrain-then-transfer) that is designed to enhance the model’s cross-domain generalization and structural awareness without introducing additional domain adaptation modules.

In the first stage, we utilize large-scale synthetic checkout images with precise instance-level mask annotations to pretrain the model. Input images are resized to 448 × 448, and the training set contains 46,000 automatically composed images. The model is trained using a batch size of 10 and an initial learning rate of 5 × 10^−4^ for a total of 60,000 iterations.

The mask branch plays a crucial role in structural guidance by supervising instance boundaries and enhancing context modeling. By leveraging mask supervision, the network learns to identify object instances precisely while capturing inter-instance structural relationships.

In the second stage, the input size is fixed at 832 × 832, and 6000 real checkout images are used for fine-tuning. A batch size of 5 is adopted, with the initial learning rate set to 1 × 10^−4^ for a total of 36,000 iterations. For evaluation on the SKU-110K dataset, we use an input size of 832 × 832, with 8233 training images and 2941 testing images. A batch size of 5 is adopted, with the initial learning rate set to 5 × 10^−4^ for a total of 50,000 iterations.

The model is initialized with the weights from the pretraining stage, but the mask branch is removed, with only the box head retained for detection on real images. This transfer process enables efficient adaptation to the target domain while preserving the structural priors learned from the synthetic data.

### 4.4. Experimental Results

To comprehensively evaluate the effectiveness of the proposed method in complex product detection scenarios, we compare it against advanced ACO methods from representative technical paradigms, including DPSNet (multi-scale detection) [51], PLACO (prototype-based alignment) [54], and DEGNet (decoupled edge guidance) [57]. These methods are selected as they represent the strongest and most competitive solutions currently available in the ACO literature. For each method, we cite the best performance reported in the original publication on the RPC dataset using the same evaluation metrics, ensuring a fair and representative comparison, as summarized in Table 2.

Our method consistently achieves the best performance across all evaluation metrics and occlusion difficulty levels, demonstrating its superior generalization and fine-grained modeling capabilities. Specifically, our method achieves cAcc of 98.52%, 97.95%, 96.52% and 97.62% under easy, medium, hard, and average mode, improving upon DEGNet by 1.32, 2.20, 3.63 and 2.37 percentage points. In localization performance, the relatively high mCIoU (99.75%) combined with low values for ACD (0.03) and mCCD (0.00) indicates stable detection and counting capability. When evaluated under COCO standards, our method shows improvements of 0.32 and 2.32 percentage points over DEGNet in mAP50 and mmAP, respectively, demonstrating consistent progress in localization accuracy.

We repeated the experiments five times. Compared with DEGNet (71.83 M parameters, 95.25 ± 0.07% cAcc), our E2MF2Net (97.88 M parameters, 97.62 ± 0.05% cAcc) shows statistically significant improvements. A paired *t*-test confirms the difference (*p* = 2.22 × 10^−16^ < 0.05), and the effect size is large (Cohen’s d = 36.74), indicating that our method exhibits distinctiveness.

**Figure 4:**
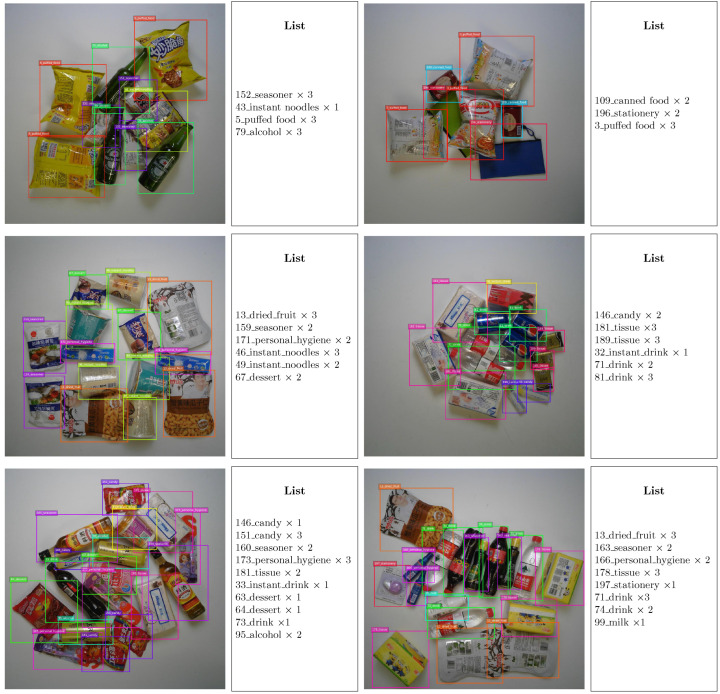
Detection results of E2MF2Net under different occlusion levels. Each row corresponds to Easy (first row), Medium (second row), and Hard (third row) modes.

As shown in Figure 4, we visualize detection results of E2MF2Net across different occlusion levels, where each row corresponds to easy, medium, and hard modes, respectively.

The results on the SKU-110K dataset are shown in Table 3. Our model achieves 42.2% mmAP and 64.5% mAP50. The performance is comparable to YOLOv7 and Deformable DETR, but lower than the task-specific DeCo-DETR. These results show that the method retains a degree of generalization even without architectural changes, tuning, or task-specific data augmentation.

### 4.5. Ablation Study

To thoroughly investigate the individual contributions of each proposed module, we conduct four groups of ablation experiments by progressively incorporating the key components: HMGC, E3, and MFF. All experiments are conducted based on the same settings and evaluation protocols to ensure fair comparisons. The results are presented in Table 4.

Experiment A, the baseline without any structural enhancement or synthetic data, achieves 93.93% cAcc under average mode, with notable drops on hard cases (90.80%) and higher ACD value (0.09). Adding HMGC in Experiment B provides modest gains in cAcc (+0.32 percentage points under average mode), as the additional generated samples improve generalization. Incorporating E3 in Experiment C further boosts performance, especially in medium and hard mode, where cAcc under hard mode increases by 3.77 percentage points over Experiment B, and ACD is reduced from 0.08 to 0.05, showing the benefit of enhanced boundary features. Most significantly, adding MFF to form the full model (Experiment D) yields the highest scores across all metrics, with average cAcc reaching 97.62%, mCIoU up to 99.75%, and minimal counting deviation (ACD = 0.03, mCCD = 0.00). These results confirm that HMGC, E3, and MFF complement each other, jointly improving classification robustness, spatial localization, and counting precision.

To comprehensively assess the impact of each component, we also report the computational costs in terms of parameter count and inference latency, as detailed in Table 5. The baseline model (without E3 or MFF) contains 83.48 M parameters and processes an image in 73.3 ms. Integrating the E3 module introduces 11.51 M additional parameters and increases latency by only 1.6 ms, indicating its efficient design for edge-aware enhancement. The subsequent inclusion of the MFF module adds a further 2.89 M parameters and 2.9 ms in latency, culminating in a full model with 97.88 M parameters and an inference speed of 77.8 ms per image. This marginal increase in computational overhead is justified by the significant gains in accuracy and robustness demonstrated in our experiments. More importantly, the final latency remains within a practical range for real-world deployment, affirming that E2MF2Net successfully balances performance with efficiency.

### 4.6. Incremental Learning

In practical ACO deployments, the continual expansion of product inventories requires detection models to support incremental learning, enabling them to quickly adapt to new product categories without forgetting previously learned classes.

To evaluate the scalability and robustness of our model under incremental learning scenarios, we conduct a three-stage evaluation simulating evolving category distributions in the RPC dataset. Following the protocol of [54], we use 183 categories as base classes and the remaining 17 categories as novel classes to ensure consistency with prior work.

In the first configuration (denoted as 183/183), the model is trained and evaluated solely on the 183 base classes. Then, in the second configuration (183/200), the same model is directly evaluated on the complete 200-class test set without further training, which allows us to assess its zero-shot generalization ability to the 17 unseen categories. Finally, in the third configuration ((183 + 17)/200), the model is incrementally fine-tuned using training samples from the 17 novel classes—while retaining data from the base classes—and subsequently evaluated on the full 200-class test set.

For comparison, we include IncreACO [50], PLACO [54] and DEGNet [57] in our evaluation as representative incremental ACO methods. The experimental results are summarized in Table 6.

The proposed method first achieves a high accuracy of 97.01% on the 183 base classes, outperforming existing approaches and demonstrating strong initial learning capability. After the incremental fine-tuning on the 17 novel categories, the model restores its overall performance to 96.59% on the full 200-class test set, showing minimal degradation on the base classes while maintaining strong generalization across all categories.

These results demonstrate that E2MF2Net exhibits strong resistance to catastrophic forgetting through a simple fine-tuning procedure, without employing any specialized continual learning techniques. This capability originates from the model’s architectural design: HMGC pre-training establishes robust structural priors, while the E3 and MFF modules enhance feature discriminability and stability. Consequently, the model adapts to new classes with minimal interference to its existing knowledge, confirming the intrinsic architectural robustness against forgetting.

## 5. Discussion

The present study introduces E2MF2Net, a unified framework designed for the ACO task. This framework simultaneously addresses key challenges in ACO, including the domain gap, fine-grained product recognition, and scalable incremental learning, by coordinating innovations at both the data and model levels. The method comprises three core components: the HMGC strategy, the MFF module, and the E3 module.

HMGC generates synthetic images that are both photorealistic and structurally consistent through pose pruning and structure-aligned composition. This process injects crucial structural priors into the learning process. More importantly, HMGC produces large-scale and structurally plausible synthetic images. These images compel the model to learn a deeper and more general understanding of product physics and occlusion. The model does not simply memorize pixel patterns. Instead, it acquires a robust and well-organized feature foundation. This foundation supports a generalized representation of checkout scenes. As a result, the features learned for base categories are less likely to be overwritten during fine-tuning. This process enhances the model’s incremental learning ability and strengthens its resistance to catastrophic forgetting. When integrated with a mask-supervised training regime, the network learns instance boundaries and spatial relationships between instances with high fidelity, significantly enhancing robustness during the transition from synthetic to real checkout images. Notably, both HMGC and the mask branch are active only during training and are entirely excluded during inference. This design ensures substantial performance improvements without introducing any additional computational cost at runtime.

The E3 module is designed to counteract the tendency of conventional feature pyramids to dilute boundary information. It leverages difference-pooling-guided, edge-aware attention to preserve fine-grained structural cues and propagate them to higher feature levels. This capability allows the model to resolve ambiguous boundaries between heavily overlapping instances, which is essential for distinguishing products under severe occlusion. Complementarily, the MFF module employs a recursive expand-compress fusion mechanism across adjacent feature levels. This process retains edge-aware details while enriching contextual semantics, thereby achieving a balance between spatial precision and semantic abstraction. As a result, MFF strengthens the discriminative feature representations necessary for fine-grained category recognition. Furthermore, the combined operation of E3 and MFF contributes to mitigating catastrophic forgetting. E3’s precise boundary modeling establishes more stable and discriminative feature representations. This reduces interference between old and new categories during fine-tuning. At the same time, MFF’s multi-scale integration enhances the robustness and generalizability of these features. As a result, they are less likely to be overwritten when learning new tasks.

Evaluated on the RPC dataset, E2MF2Net outperforms current state-of-the-art methods. The model attains cAcc of 98.52%, 97.95%, 96.52%, and 97.62% in the Easy, Medium, Hard, and Average mode, respectively, and yields a notable improvement of 3.63 percentage points in the Hard mode relative to a strong baseline (DEGNet). Given that ACO emphasizes complete and accurate shopping-list generation, instance-level counting and categorical correctness (i.e., cAcc) serve as the primary evaluation criteria. Although COCO-style metrics such as mAP50 and mmAP are treated as supplementary diagnostics, the observed improvements in these metrics further corroborate enhanced localization accuracy.

Ablation studies validate the contribution of each component, and incremental-learning experiments demonstrate that the model experiences only minor degradation after the inclusion of novel classes and can rapidly recover via fine-tuning. We acknowledge that existing continual learning methods offer effective mechanisms for mitigating catastrophic forgetting. However, these methods often impose additional constraints and introduce significant computational overhead. In contrast, our framework achieves competitive incremental learning performance through architectural innovations and pre-training strategies alone, without relying on explicit continual learning techniques. The observed resistance to forgetting indicates that, in application domains such as ACO, carefully designed pre-training and feature representation strategies can substantially reduce the need for complex continual learning algorithms.

Despite the encouraging results, several limitations of the proposed method remain and suggest directions for future work. A first limitation lies in the computational cost of HMGC. Its $O(n^{2})$ mask and edge operations become heavy when processing high-resolution images. This is manageable for checkout-counter images with fixed sizes (e.g., 1815 × 1815), but scaling to larger scenes is challenging. In addition, HMGC works well for rigid products but struggles with deformable items such as soft packages. It also has difficulty modeling extreme occlusions. These issues are caused by its reliance on 2D composition and the limited diversity of available single-product captures.

Dataset reliance is another constraint. RPC, while the largest and most representative ACO benchmark, focuses only on checkout-counter layouts. This narrow scope limits evaluation diversity. Results on SKU-110K show that our method does not fully adapt to shelf-style settings, which contain dense arrangements and many small objects. The absence of specialized post-processing and dataset-specific augmentation further reduces performance in such cases. Moreover, most ACO datasets remain unimodal, relying only on visual information. This prevents the model from exploiting complementary signals such as depth sensing or RFID.

While the proposed method achieves competitive accuracy, we acknowledge certain limitations in computational efficiency. The main constraints on parameters and inference speed arise from the complexity of the MViTv2 backbone and the Cascade R-CNN framework. Notably, the HMGC strategy operates only during the data generation phase and does not introduce any overhead at inference. In addition, the E3 and MFF modules contribute only marginally to the total parameter count and inference latency, as confirmed by our ablation studies.

Future work will address these challenges. One direction is to integrate 3D-aware modeling or physics-based simulation into HMGC, improving the handling of deformable items and occlusion. Another is to combine the framework with continual learning to support growing product catalogs while avoiding catastrophic forgetting. Deployment efficiency could also be improved through lighter backbones, knowledge distillation, pruning, and quantization. Furthermore, we plan to improve dense small object detection by optimizing post-processing components, including NMS strategies and IoU-based loss functions. These enhancements aim to boost generalization across datasets with different object distributions and scales. Finally, multimodal inputs and domain adaptation strategies should be explored to strengthen robustness in heavily occluded or diverse retail environments.

## Figures and Tables

**Table 1:** Statistics of the RPC and SKU-110K datasets.

Datasets	Train_Image	Test_Image	Total_Image
RPC	46,000 (synthetic) + 6000 (real)	24,000 (real)	76,000
SKU-110K	8233	2941	11,174

**Table 2:** Performance comparison with state-of-the-art ACO methods on the RPC dataset under easy, medium, hard, and average mode.

Network	${\mathrm{cAcc}}_{easy}$	${\mathrm{cAcc}}_{medium}$	${\mathrm{cAcc}}_{hard}$	${\mathrm{cAcc}}_{average}$	mCIoU	ACD	mCCD	mAP50	mmAP
DPSNet [51]	97.22%	91.64%	85.06%	90.74%	99.12%	0.10	0.01	99.02%	82.72%
PLACO [54]	95.97%	94.73%	90.47%	93.73%	99.20%	0.10	0.01	98.94%	85.62%
DEGNet [57]	97.20%	95.75%	92.89%	95.25%	99.47%	0.07	0.01	99.02%	84.96%
**Ours**	**98.52%**	**97.95%**	**96.52%**	**97.62%**	**99.75%**	**0.03**	**0.00**	**99.34%**	**87.28%**

The proposed method achieves the highest scores across all evaluation metrics, consistently outperforming strong baselines in both classification and localization accuracy, with particularly large gains under the hard mode. Bold numbers indicate the best performance.

**Table 3:** Performance Comparisons on the SKU-110K dataset.

Network	mmAp	mAP50
Faster R-CNN	38.4%	62%
YOLOv7	42.6%	62.3%
DERT	25.6%	37.8%
Deformable DERT	42.6%	63.5%
DAB-DERT	33.9%	81%
DeCo-DETR	54.8%	93.3%
Ours	42.2%	64.5%

All results of other models are reported from [67].

**Table 4:** Ablation studies of proposed method on the RPC dataset.

Experiment	HMGC	E3	MFF	${\mathrm{cAcc}}_{easy}$	${\mathrm{cAcc}}_{medium}$	${\mathrm{cAcc}}_{hard}$	${\mathrm{cAcc}}_{average}$	mCIoU	ACD	mCCD
A				96.21%	94.95%	90.80%	93.93%	99.30%	0.09	0.01
B	✔			96.65%	95.00%	91.31%	94.25%	99.35%	0.08	0.01
C	✔	✔		97.72%	96.78%	95.08%	96.49%	99.61%	0.05	0.00
D	✔	✔	✔	**98.52%**	**97.95%**	**96.52%**	**97.62%**	**99.75%**	**0.03**	**0.00**

Notes: HMGC refers to a data generation method combined with corresponding training strategies. Experiments without HMGC (e.g., Experiment A) are trained directly on 6000 real checkout images. A checkmark (✔) indicates that the corresponding component is included in the experiment. Bold numbers indicate the best performance.

**Table 5:** Ablation studies about computational costs of proposed method.

E3	MFF	Params	Inference
		83.48 M	73.3 ms/img
✔		94.99 M	74.9 ms/img
✔	✔	97.88 M	77.8 ms/img

**Table 6:** Comparison of incremental learning with other methods.

Network	Train/Test	${\mathrm{cAcc}}_{average}$
IncreACO [50]	183/183	78.76%
	183/200	47.53%
	(183 + 17)/200	74.3%
PLACO [54]	183/183	89.25%
	183/200	47.35%
	(183 + 17)/200	87.39%
DEGNet [57]	183/183	91.97%
	183/200	50.86%
	(183 + 17)/200	91.35%
Ours	183/183	97.01%
	183/200	53.42%
	(183 + 17)/200	96.59%

## Data Availability

The original data presented in the study are openly available in [Retail Product Checkout Dataset] at [https://rpc-dataset.github.io] (accessed on 1 April 2024).
